# Patterns of Dermatological Diseases in Inpatient Consultations at King Abdulaziz Medical City, Jeddah, Saudi Arabia: An Underexploited Opportunity for Dermatology Clinical Training

**DOI:** 10.7759/cureus.22132

**Published:** 2022-02-11

**Authors:** Awadh Alamri, Mohammed Alshareef, Sarah B Aljoudi, Luai Assaedi, Sara Alkassimi, Abdulmohsin Algethami, Mazen S Dajam

**Affiliations:** 1 Dermatology Department, College of Medicine, King Saud Bin Abdulaziz University for Health Sciences, King Abdulaziz Medical City, Jeddah, SAU; 2 Dermatology Department, King Abdulaziz Medical City, Jeddah, SAU

**Keywords:** saudi, consultation, referral, inpatient, skin disorders, dermatology

## Abstract

Background

Inpatient dermatological care represents an opportunity to improve dermatological care among the population as well as to enhance clinical exposure for residents and medical trainees.

Objective

We conducted this study to analyze the pattern of dermatological conditions encountered in inpatient settings and the modalities of management at a tertiary care hospital.

Method

We retrospectively reviewed and analyzed electronic records of all inpatient consultations carried out by the dermatology consultants and specialists between January 1, 2020 and December 31, 2020. Demographic and specific and non-specific clinical data were collected and analyzed by dividing the skin disorders and treatments into categories, where relevant.

Result

Five hundred and seventy-one inpatient dermatological consultations were carried out, involving 453 patients. Older age groups were predominant, including 50-70 years (27.4%) and >70 years (21.0%). The female to male ratio was 1.19. The majority of the consultations (388/571, 68.1%) were requested from the adult medical wards; internal medicine (23.8%), hematology (13.7%), and oncology (9.1%) being the most frequented wards. A biopsy was carried out in 57 (10.0%) of the cases. The most prevalent diagnoses included dermatitis (16.3%), intertrigo (8.1%), and xerosis (6.8%). Besides, 10 cases of skin cancer or metastasis were diagnosed by the dermatologist. The diagnosed skin condition was drug-induced in 57 (10.0%) of the cases, and nine of them were due to chemotherapy. Pharmaceutical treatments consisted of more frequently used corticosteroids (51.5%), antibiotics (36.4%), and antifungal agents (20.8%), with the majority of these by topical route.

Conclusion

A broad range of dermatological conditions are diagnosed in our inpatient setting, representing a good educational opportunity for trainee dermatologists. The implementation of digital photography could enhance the documentation of dermatological conditions, which would have beneficial effects on both care quality and education.

## Introduction

Skin is the largest organ of the body and is subjected to plentiful pathological manifestations with variable etiological and prognostic profiles [1]. Hospital-based dermatological care represents a valuable asset for hospitalized patients, enabling timely diagnosis and management of severe and life-threatening conditions such as purpura fulminans, Stevens-Johnson syndrome, or drug adverse reactions [2,3]. The inpatient setting constitutes an opportunity for extended investigations of skin conditions and provides access to better care and follow-up, enhancing both the diagnosis and the outcome of the skin lesions, besides the patient education [4]. Such an opportunity could be more perceptible among patients with low socioeconomic status or living in disadvantageous areas, which may increase the burden of inpatient dermatology on the healthcare system. A national US study showed that a dermatological condition was diagnosed among one in eight adults who were hospitalized in 2014, resulting in an estimated cost of five billion dollars [5].

On the other hand, the inpatient environment contributes to the clinical training of physicians, notably regarding uncommon dermatological diseases [6]. In Saudi Arabia, it was estimated that the healthcare force comprises fewer than four dermatologists for 100,000 inhabitants [7], while other local data showed low levels of satisfaction among residents from the dermatology residency program about the clinical training in dermatology, especially with regards to certain procedures [8]. This emphasizes the importance of further improving the inpatient dermatology care to enhance clinical exposure among the trainees.

This study was conducted to provide insights into the pattern of dermatological conditions encountered in inpatient settings and the modalities of management. Analysis of such data enables improving the quality of care in dermatology by enhancing collaboration with non-dermatologist healthcare teams and offering educational guidance to approach the most prevalent conditions.

## Materials and methods

We retrospectively reviewed and analyzed electronic records of all inpatient consultations carried out by the dermatology consultants and specialists at King Abdulaziz Medical City (KAMC), Jeddah, Saudi Arabia, between January 1, 2020 and December 31, 2020. The study was ethically approved by the institutional review board of KAIMRC (reference number: JED-21427780-31707).

An excel sheet was used to collect the following data: demographic data; patient identifier number; significant medical history; date of consultation; department of referral; dermatological lesion description and location; any investigations (biopsy, culture, etc.); final diagnosis; and management, including topical and systemic treatments, as well as non-pharmaceutical treatments, such as patient education.

Lesion locations were categorized into: oral, face, scalp, back, trunk and axillary region, upper limb, lower limbs, hands and palms, feet, soles and toes, pelvis and anogenital region, and generalized. Diagnoses were, to the extent possible, grouped into categories, such as bacterial infections (impetigo, cellulitis, etc.), cancers and metastasis, acneiform eruptions (acne vulgaris, rosacea, and folliculitis), fungal infections, vasculitis, etc. Treatments were categorized into pharmaceutical and non-pharmaceutical treatments. Among pharmaceutical treatments, corticosteroids, antibiotics, and antifungal agents were categorized into systemic and topical.

The data were checked, cleaned, and coded in Microsoft Excel (Microsoft Corporation, Redmond, Washington, USA), then transferred to the Statistical Package for Social Sciences version 21.0 for Windows (SPSS Inc., Chicago, IL, USA). Frequencies and percentages were calculated.

## Results

Consultations and patients’ characteristics

Five hundred and seventy-one inpatient dermatological consultations were carried out between January 1, 2020 and December 31, 2020, involving 453 patients. Patients’ characteristics are presented in Table 1, showing the predominance of older age groups, including 50-70 years (27.4%) and >70 years (21.0%). The female to male ratio was 1.19. The most frequent comorbidities were diabetes mellitus (30.9%), hypertension (29.8%), and cardiac diseases (14.1%).

**Table 1:** Patients’ characteristics (N=453). Gender was not specified for one patient. § A patient may have more than one comorbidity. ESRD: end stage renal disease; SLE: systemic lupus erythematosus.

Parameter	Category	Frequency	Percentage
Age category	0-30 days	4	0.9
	>1-24 months	18	4.0
	>2-5 years	21	4.6
	>5-12 years	26	5.7
	>12-19 years	26	5.7
	>19-30 years	46	10.2
	>30-50 years	91	20.1
	>50-70 years	124	27.4
	>70 years	95	21.0
	Unspecified	2	0.4
Gender	Male	206	45.6
	Female	246	54.3
No. of consultations by patient	1	369	81.5
	2	30	13.2
	3	17	3.8
	4+	7	1.5
Comorbidities §	Diabetes mellitus	140	30.9
	Hypertension	135	29.8
	Cardiac disease	64	14.1
	Liver cirrhosis	11	2.4
	ESRD	37	8.2
	Hypothyroidism	24	5.3
	Asthma	24	5.3
	SLE	10	2.2
	Cerebrovascular accident	26	5.7
	Malignancy	178	39.3
	Bowel inflammatory disease	11	2.4
	COVID-19	7	1.5
	Other	146	32.2

The majority of the consultations (388/571, 68.1%) were requested from the adult medical wards; internal medicine (23.8%), hematology (13.7%), and oncology (9.1%) being the most frequented wards. Patients from surgical wards and pediatric wards represented 15.8% and 15.6% of the total consultations, respectively (Table 2).

**Table 2:** Referral departments (N=571 consultations). ICU: intensive care unit; ENT: ear nose and throat.

Department	Frequency	Percentage
Adult medial wards	388	68.1
Internal medicine	136	23.8
Hematological oncology	78	13.7
Oncology	52	9.1
Obstetrics-gynecology	26	4.6
Cardiology	15	2.6
Neurology	14	2.5
Emergency	13	2.3
Gastroenterology	11	1.9
ICU	10	1.8
Nephrology	9	1.6
Other medical wards	24	4.2
Adult surgical wards	90	15.8
Orthopedics	19	3.3
Vascular surgery	17	3.0
ENT	14	2.5
Neurosurgery	13	2.3
General surgery	8	1.4
Ophthalmology	3	0.5
Urology	3	0.5
Other surgical wards	13	2.3
Pediatric wards	89	15.6
Pediatric hematology-oncology	41	7.2
General pediatrics	31	5.4
Other pediatric wards	11	1.9
Pediatric surgery	6	1.1
Not documented	4	0.7

Clinical and paraclinical investigations

Details of dermatological examination findings were documented in 5.3% of the cases regarding the type of lesion and 15.6% of the cases regarding the location. A biopsy was carried out in 57 (10.0%) of the cases, a culture in 69 (12.1%), and blood tests were requested in 34 (6.0%) (Table 3). Other investigations included venous Doppler, bone marrow, pathergy test, genetic testing, and cytopathology, in one patient each.

**Table 3:** Dermatological examination data (N=571 consultations). § A patient may have more than one location.

Parameter	Category	Frequency	Percentage
Lesion description	Bulla	6	1.1
	Macule	7	0.2
	Papule	1	0.2
	Ulcer	3	0.4
	Desquamation	3	0.5
	Rash	6	1.1
	Petechia	1	0.2
	Erythema	3	0.5
	Vesicle	1	0.2
	Unspecified skin lesion	4	0.7
	Allergy	1	0.2
	Not documented	541	94.7
No. of lesions	1	28	4.9
	2	2	0.4
	Not documented	541	94.7
Location of lesions §	Oral	6	1.1
	Face/ear	13	2.3
	Scalp/occiput	12	2.1
	Back	7	1.2
	Trunk/axilla	15	2.6
	Upper limb	7	1.2
	Lower limb	13	2.3
	Hand/palm	13	2.3
	Foot/sole/toe	11	1.9
	Pelvis/anus/genital organ	34	6.0
	Generalized	4	0.7
	Not documented	482	84.4
No. of localizations	1	58	10.2
	2	19	3.3
	3+	12	2.2
	Not documented	482	84.4
Investigations	Biopsy	57	10.0
	Culture	69	12.1
	Blood tests	34	6.0
	Other	4	0.7

Final diagnoses

The final diagnosis was documented in 87.4% of the inpatient consultation reports, and more than one diagnosis was reported in 21.2% of the cases. The most prevalent diagnoses included dermatitis (16.3%), intertrigo (8.1%), and xerosis (6.8%). Of note, 10 cases of skin cancer or metastasis were diagnosed by the dermatologist. The diagnosed skin condition was drug-induced in 57 (10.0%) of the cases, and nine of them were due to chemotherapy (Table 4).

**Table 4:** Final dermatological diagnoses among inpatient consultations (N=571). § A patient may have more than one diagnosis.

Parameter	Category	Frequency	Percentage
No. of diagnoses	Unspecified	72	12.6
	1	378	66.2
	2	101	17.7
	3	18	3.2
	4	2	0.4
Diagnosis §	Dermatitis	93	16.3
	Intertrigo	46	8.1
	Xerosis/dry skin	39	6.8
	Secondary to other condition	35	6.1
	Other eruption (non-specified)	30	5.3
	Fungal infection	29	5.1
	Acneiform eruptions	23	4.0
	Bacterial infection	22	3.9
	Eczema	19	3.3
	Non-specific skin reaction	18	3.2
	Herpes zoster	15	2.6
	Urticaria/allergic eruption	17	3.0
	Bullous disease	14	2.5
	Ulcer	13	2.3
	Scars	11	1.9
	Vasculitis	10	1.8
	Psoriasis	10	1.8
	Skin metastasis of cancer	10	1.8
	Candidiasis	9	1.6
	Warts	8	1.4
	Parasite	7	1.2
	Dermatosis	4	0.7
	Ecchymosis	3	0.5
	Other	153	26.8
Drug-induced	Yes	57	10.0
	No	514	90.0

Cases that were diagnosed with biopsy are presented in Table 5. These included 8 (14.0%) cases of bullous diseases, of which 7 (12.3%) were bullous pemphigus, 8 (14.0%) cases of skin metastasis or cancer, and 6 (10.5%) cases of vasculitis.

**Table 5:** Dermatological diseases diagnosed by biopsy (N=57).

Disease	Frequency	Percentage
Bullous disease	8	14.0
Bullous pemphigoid	7	12.3
Immunobullous dermatosis	1	1.8
Skin metastasis of cancer	8	14.0
Metastatic alveolar soft part sarcoma	1	1.8
Poorly differentiated carcinoma	1	1.8
Secondary cutaneous metastasis	1	1.8
Metastatic adenocarcinoma	1	1.8
Anaplastic large cell lymphoma	1	1.8
Cutaneous metastasis of patients known peripheral t-cell lymphoma	1	1.8
Cutaneous lymphoma	1	1.8
Pigmented basal cell carcinoma	1	1.8
Vasculitis	6	10.5
Henoch-Schonlein purpura	2	3.5
Small vessel vasculitis	2	3.5
Leukocytoclastic vasculitis	1	1.8
Urticarial vasculitis	1	1.8
Allergic contact dermatitis	2	3.5
Disseminated fungal infection	2	3.5
Graft versus host disease	2	3.5
Lipoma	2	3.5
Allergic contact dermatitis	2	3.5
Acquired perforating dermatosis	2	3.5
Acute generalized exanthematous pustulosis	1	1.8
IgA pemphigus	1	1.8
Drug eruption	1	1.8
Superficial peri vascular lymphatic infiltrate	1	1.8
Angular stomatitis	1	1.8
Pustular dermatosis	1	1.8
Suppurative folliculitis	1	1.8
Psoriasis	1	1.8
Angular cheilitis	1	1.8
Calcinosis cutis	1	1.8
Calciphylaxis	1	1.8
Ecthyma gangrenousum	1	1.8
Erythema multiforme	1	1.8
Histiocytic infiltrate	1	1.8
Lupus	1	1.8
Lymph node	1	1.8
Reactive lymphoid hyperplasia	1	1.8
Sweet's syndrome	1	1.8
Vasculopathy	1	1.8
Viral exanthema	1	1.8
Wound	1	1.8
Calcinosis cutis	1	1.8
Calciphylaxis	1	1.8
Cutaneous lymphoma	1	1.8

Of the drug-induced cases (N=57), the most prevalent included unspecific eruptions (22, 38.6%), urticarial or anaphylactic (16, 28.1%), and morbilliform drug reactions (10.5%), and 9 of them were toxic erythema of chemotherapy (results not presented in tables).

Management

Pharmaceutical treatment was prescribed in 84.8% of the cases. Corticosteroids were prescribed in 51.5% of the cases, including 4.0% by systemic route. Antibiotics were prescribed in 36.4% of the cases, including 2.3% by systemic route. Antifungal agents were prescribed in 20.8% of the cases, including 1.4% by systemic route. Other frequently prescribed agents were antihistamines (22.8%), emollients (18.6%), and moisturizers (18.0%). Patient education was indicated in 28.5% of the cases, and patients were referred in 10.7% of the cases (Table 6).

**Table 6:** Management of dermatology cases among inpatient consolations (N=571). § A patient may have more than one treatment.

Parameter	Category	Frequency	Percentage
No. of pharmaceutical treatments	0	87	15.2
	1	127	22.2
	2	158	27.7
	3	115	20.1
	4	59	10.3
	5+	25	4.4
Pharmaceutical treatments §	Corticosteroids	294	51.5
	Systemic	23	4.0
	Topical	276	48.3
	Antibiotics	208	36.4
	Systemic	13	2.3
	Topical	194	36.4
	Antifungal	119	20.8
	Systemic	8	1.4
	Topical	107	18.7
	Shampoo	18	3.2
	Antihistamine	130	22.8
	Emollient	106	18.6
	Moisturizer	103	18.0
	Potassium permanganate	84	14.7
	Zinc oxide	49	8.6
	Wound care	38	6.7
	Antiviral	20	3.5
	Cryotherapy	4	0.7
Other approaches	Education	163	28.5
	Referral	61	10.7
	Reassurance	33	5.8

## Discussion

This single-center retrospective review conducted during the year 2020 showed that 571 dermatological consultations were provided in an inpatient setting, corresponding to an average of 47 consultations per month. During the COVID-19 crisis, inpatient dermatology care was highly solicited due to reduced outpatient care, notably during the lockdown. Although most of the skin manifestations were benign, a non-negligible percentage were potentially life-threatening especially, in immunocompromised patients such as those in palliative care, intensive care, or patients on chemotherapy.

Internal medicine represented the main client of inpatient dermatology care, followed by hematology and oncology, both adult and pediatric. This is consistent with several studies and probably reflects the relatively high patient flow in these departments, besides the particular patterns of diseases [9-11].

The most prevalent group of skin manifestations was represented by dermatitis, accounting for 16.3% of the consultations. Most of these cases were benign conditions such as contact dermatitis or seborrheic dermatitis. This is consistent with a study by Neloska et al., which showed that dermatitis represented 18.3% of the consultations in palliative care [12]. Likewise, a study from Singapore showed eczematous dermatitis to be the second most frequent dermatological condition found in hematology wards, accounting for 13.3% of the consultations [13]. Consistently, an Irish study showed atopic dermatitis to be the second most prevalent condition in 12% of the consultations [14]. Such observations suggest the relevance of training non-dermatologist physicians in the diagnosis and management of the most common benign dermatological conditions.

Cutaneous bacterial and fungal infections were diagnosed in 3.9% and 5.1%, respectively, with the possibility of two concomitant infections in one patient. However, antibiotics and antifungal agents were prescribed in 36.4% and 20.8%, but only 2.3% and 1.4% were by systemic routes, respectively. This demonstrates that topical antimicrobial agents are often adequate for cutaneous infections. On the other hand, the high percentage of topical use, compared with the number of infections, indicates the large use of topical antibiotics and antifungal agents, notably to prevent wound infection among immunocompromised patients. In contrast to our findings, cutaneous infections were reported to be the second most frequent diagnosis in a pediatric hematology ward in Riyadh, as observed by Alasmari et al., representing 13.3% of the consultations [15]. In Italy, a single-center study from a university hospital also showed infections to be the most prevalent cause of inpatient consultations in dermatology (27.1%) [1]. Another study in hematology, from Singapore, observed cutaneous infections to be the leading motivation of dermatology inpatient consultations, representing 15.0% of the total consultations [13]. This is consistent with the data from an Irish hospital showing cutaneous infections to be the main group of conditions diagnosed for inpatient referrals to dermatology, with 22.0% of the consultations [14].

Drug-induced manifestations represented 57 (10.0%) of the total consultations, nine of which were due to toxic erythema of chemotherapy. There are several life-threatening drug-induced reactions that have a dermatological presentation. Among these are Stevens-Johnson syndrome and toxic epidermal necrolysis, which can be lethal in one-third of the cases and require timely diagnosis and management. The other frequent condition consists of drug eruption with eosinophilia, which is due to hypersensitivity [2]. Toxic erythema of chemotherapy represents a heterogeneous group of dermatological manifestations with often challenging diagnoses. They can present as painful erythema or bullous dermatosis, as they may mimic other conditions such as intertrigo, contact dermatitis, or hypersensitivity reactions [16].

One of the major observations in the present study is the lack of dermatological examination data in the majority of cases, which highlights the need to improve practice. It is, however, essential to analyze the factors associated with such an issue. One of the parameters to consider is the relatively large number of consultations requested, which may constitute an overload for the dermatology team, besides their routine visits and outpatient clinics. Another parameter is the COVID-19 pandemic, which represents an additional workload. As such, it is recommended to implement organizational measures to improve the performance of hospital-based dermatologists, notably during COVID-19. The use of telehealth technology would have the double advantage of improving access to dermatological care and enabling record-keeping of photographs of dermatological lesions. Indeed, teledermatology has a great interest in triaging and identifying dermatological conditions [17]. Additionally, the use of telemedicine in inpatient dermatology can have an educative effect on both dermatology and non-dermatology physicians [18]. On the other hand, the use of digital photography may be the best strategy to document dermatological conditions; it enables saving time and offers the possibility of cross-diagnosis, in addition to comparative analysis of the progression of the skin lesions [19].

Limitations

The present study is principally limited by the retrospective design, resulting in high information bias as shown by the lack of dermatological examination data.

## Conclusions

A broad range of dermatological conditions are diagnosed in our inpatient setting, representing a good educational opportunity for trainee dermatologists. The use of digital photography may be of great interest to enhance the documentation of dermatological conditions in an inpatient setting, which would have beneficial effects both on patient care and physicians’ education. Comprehensive strategies can be implemented to enhance the organizational aspects of inpatient dermatology care to improve both care and training quality, notably during the COVID-19 pandemic.
